# Spontaneous Regression of Pulmonary Metastases from Breast Angiosarcoma

**DOI:** 10.1155/2008/940656

**Published:** 2008-12-03

**Authors:** S. W. Kim, J. Wylie

**Affiliations:** Department of Clinical Oncology, Christie Hospital NHS Trust, Wilmslow Road, Withington, Manchester M20 4BX, UK

## Abstract

Spontaneous regression of cancer is a rare phenomenon. We present a rare case of pulmonary metastases in a 72-year-old woman with metastatic breast angiosarcoma. She was diagnosed with a breast angiosarcoma in 2005 and underwent a total mastectomy and postoperative radiotherapy. Unfortunately, a year later she was found to have multiple lung and scalp metastases but in a view of her poor general fitness, she was not a candidate for chemotherapy and was kept on regular followup. Despite the absence of any treatment, the followup chest X-ray showed a significant reduction in the number and size of lung nodules and her scalp lesions regressed completely. Seven months after the diagnosis of metastatic disease, the nodules in her scalp remain controlled.

## 1. INTRODUCTION

Spontaneous regression (SR) of cancer is a rare but well-known phenomenon, which has been reported in several types of human cancer. It has been most commonly reported in carcinoma of the kidney, neuroblastoma, malignant melanoma, and choriocarcinoma [1]. We present a case of spontaneous regression of pulmonary metastases from a primary breast angiosarcoma.

## 2. CASE REPORT

**Figure 1:**
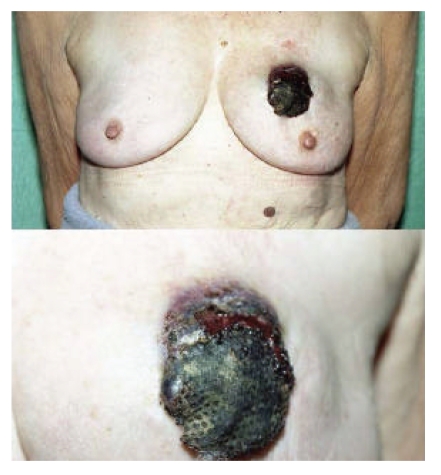
An ulcerated bruise-like lesion in the left breast, consistent with the typical appearance of a breast angiosarcoma.

A 72-year-old lady presented to a local emergency department with an ulcerating bruised lesion in her left breast (Figure 1). A true-cut biopsy of the mass confirmed an angiosarcoma and she subsequently underwent a left simple mastectomy in June 2005. The final histology showed a 40 mm ulcerated lesion showing the typical features of an epithelioid angiosarcoma. The tumour was predominantly well differentiated with smaller areas showing poorly differentiated tumour cells. The tumour was excised completely but the closest posterior margin was only 1 mm. Following referral to our institution, postoperative radiotherapy was delivered to the chest wall using a single 8 Mev electron field, delivering 40 Gy in 15 fractions over 3 weeks followed by an electron boost to the scar of 10 Gy in 4 fractions. A staging CT thorax and abdomen at this time showed no evidence of distant metastases.

**Figure 2:**
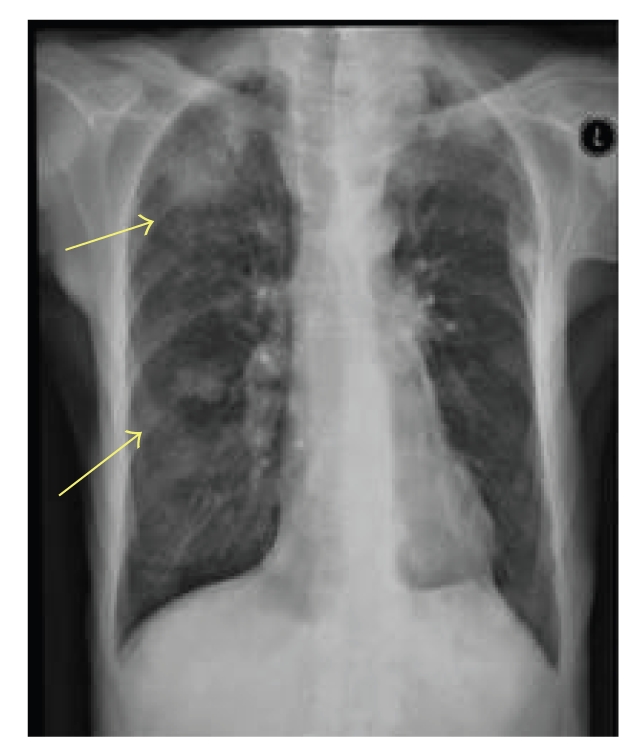
Chest radiograph showing multiple lung metastases.

Unfortunately, in May 2006, the patient presented to the clinic with symptoms of shortness of breath and a chest radiograph revealed multiple lung metastases. A subsequent CT scan showed multiple bilateral lung nodules considered too numerous to consider metastectomy. Due to the patient's poor general fitness, she was not a candidate for chemotherapy but was kept on regular follow-up. Over the next few months, she developed symptoms of disease progression and the repeated chest X-rays showed an increase in the size and number of lung nodules (Figure 2). Physical examination at that time also revealed multiple new subcutaneous nodules on her scalp consistent with metastatic skin deposits. As these nodules were not causing her any symptoms, no palliative treatment was offered at that time.

**Figure 3:**
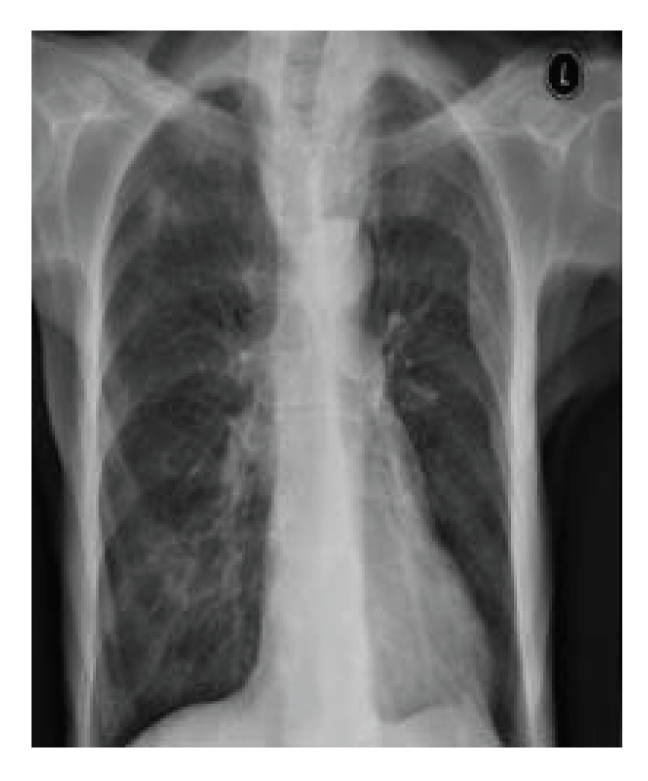
Repeat chest radiograph performed three months later showing a significant reduction in size and number of lung metastases.

She was reviewed again in the clinic in December 2007 and commented that the scalp nodules had regressed. On clinical examination, the subcutaneous scalp nodules were no longer palpable and a subsequent chest X-ray showed a reduction in the number and size of lung nodules. The patient denied taking any nonprescribed medication or dietary change leading to the conclusion that this represented a spontaneous regression (Figure 3).

## 3. DISCUSSION

Primary sarcoma of the breast is a rare malignant tumour which accounts for approximately 0.04% of all breast tumours [2]. It typically presents as a palpable breast mass and the highly vascular nature of these tumours often produces a bluish discoloration of the overlying skin. The discoloration is often initially mistaken for bruising, thereby delaying diagnosis. The primary treatment is surgical excision, which often requires a simple mastectomy in order to obtain wide margins. A routine axillary dissection is not indicated as lymphatic spread is uncommon (although more common than many other sarcomas) [3].

There has been an increased incidence of soft tissue sarcomas in breast cancer patients who underwent radiotherapy treatments [4–6]. One study reported 16-fold increased risk of angiosarcoma and 2-fold increased risk of other sarcomas in postradiotherapy patients [6].

A positive surgical margin is an important risk factor for disease recurrence, and the use of adjuvant radiotherapy has shown to be related to reduce local recurrence [7]. Although radiotherapy has not shown to improve survival, given the well-known benefit of radiotherapy in soft tissue sarcomas at other tumour sites, it is reasonable to offer postoperative radiotherapy if there is high risk of microscopic residual disease [7]. The prognostic factors for sarcoma of the breast include the tumour grade, size, presence of residual disease, and cellular pleomorphism [8, 9]. There is no definite evidence to support the use of adjuvant chemotherapy in angiosarcoma [9].

Spontaneous regression (SR) of cancer is defined as a partial or complete disappearance of a malignant tumour in the absence of medical treatment or in the presence of treatment which is considered to be inadequate to produce a significant influence on tumour regression [10]. Several mechanisms have been proposed to explain SR of cancer. These include stimulation of an immune response, the elimination of carcinogens, angiogenesis inhibition, hormonal mediation, enhanced apoptosis, and epigenetic mechanisms [11].

In normal endothelial cells, there is a balance between endogenous proangiogenic factors and antiangiogenic factors. An increased expression of proangiogenic factor, such as vascular endothelial growth factor (VEGF), can be seen in cancer cells leading to the uncontrolled growth of blood vessels promoting the further growth of tumour cells. Angiostatin, a naturally occurring antiangiogenic factor, has shown to suppress the tumour growth in several animal studies [12], and there is experimental evidence showing the increased growth of micrometastases when endogenous inhibition of angiogenesis reduces following resection of the primary tumour [13]. An activation of intrinsic antiangiogenic factors may play a role in spontaneous regression of cancer but an exact explanation of this remains an area for research.

The immune system can be stimulated by several different factors including bacterial or viral infections, hormonal influences, and trauma. In the original report on SR of cancer by Everson and Cole, 71 patients had undergone an operation and 8 had experienced infections before showing spontaneous regression [14]. Since Cole (1981), there have been numerous reports on spontaneous tumour regression and many of them described the association between SR and concomitant infections [15].

In the early part of 1900 William Coley, a surgeon from New York, reported tumour regression in patients following a streptococcal infection of an ulcerated tumour. He subsequently showed that the immune system could be stimulated through the administration of a vaccine consisting of killed gram-positive *Streptococcus pyogenes* and gram-negative *Serratia marcescens,* the so-called “Coley's toxins” [16]. Coley showed that the induction of a mild-to-moderate fever was necessary to stimulate the immune system sufficient to produce tumour regression. Although Coley's toxins are no longer routinely prescribed in standard cancer management, a similar methodology explains the response to superficial bladder cancer following the intravesicle administration of BCG [11]. Later studies have shown that the SR of cancer following infection may be explained by the release of cytokine and associated cellular immune reaction results in inflammatory necrosis or T cell-mediated apoptosis [17].

Changes in the activity of normal genes are well known to be responsible for the development of cancer, and, as a result, any alterations in DNA methylation can contribute to the malignant transformation of the cells [18]. Studies in genetic alterations in carcinogenesis showed the frequent involvement of genes that are inactivated by hypermethylations with tumours that often undergo spontaneous regression [19]. An increased response of tumour cells to the apoptotic stimuli is also involved in the process of SR as well as the activation of host immune response involving cytokines such as interleukin and interferon gamma [20].

Spontaneous regression of pulmonary metastases from an angiosarcoma is a rare event. A medline search found similar reports on spontaneous regression of lung metastases in patients with osteosacoma [21] and endometrial stromal sarcoma [22] but there were no reports on SR in angiosarcoma. Our patient had no history of recent trauma or infection to suggest a stimulation of her immune system as an explanation of this unusual phenomenon. Seven months after her diagnosis with metastatic disease, the patient remains stable without receiving any form of treatment and the subcutaneous nodules in her scalp remain controlled.

In conclusion, spontaneous regression of lung metastases in breast angiosarcoma is rare and its mechanism remains uncertain. Further understanding of the exact pathways of immune activation may help the development of anticancer treatments.
